# Evaluation of pulse wave transit time analysis for non-invasive cardiac output quantification in pregnant patients

**DOI:** 10.1038/s41598-020-58910-x

**Published:** 2020-02-05

**Authors:** Emmanuel Schneck, Pascal Drubel, Rainer Schürg, Melanie Markmann, Thomas Kohl, Michael Henrich, Michael Sander, Christian Koch

**Affiliations:** 1 Justus Liebig University of Giessen, Department of Anesthesiology, Operative Intensive Care Medicine and Pain Therapy, Rudolf-Buchheim-Strasse 7, 35392 Giessen, Germany; 2 German Center for Fetal Surgery & Minimally Invasive Therapy (DZFT), University Hospital of Mannheim, Theodor-Kutzer-Ufer 1-3, 68167 Mannheim, Germany; 3 Department of Anesthesiology and Intensive Care Medicine, St. Vincentius Clinics, Suedendstrasse 32, 76137 Karlsruhe, Germany

**Keywords:** Diagnosis, Clinical trial design

## Abstract

Pregnant patients undergoing minimally-invasive foetoscopic surgery for foetal spina bifida have a need to be subjected to advanced haemodynamic monitoring. This observational study compares cardiac output as measured by transpulmonary thermodilution monitoring with the results of non-invasive estimated continuous cardiac output monitoring. Transpulmonary thermodilution-based pulse contour analysis was performed for usual anaesthetic care, while non-invasive estimated continuous cardiac output monitoring data were additionally recorded. Thirty-five patients were enrolled, resulting in 199 measurement time points. Cardiac output measurements of the non-invasive estimated continuous cardiac output monitoring showed a weak correlation with the corresponding thermodilution measurements (correlation coefficient: 0.44, R^2^: 0.19; non-invasive estimated continuous cardiac output: 7.4 [6.2–8.1]; thermodilution cardiac output: 8.9 [7.8–9.8]; *p* ≤ 0.001), while cardiac index experienced no such correlation. Furthermore, neither stroke volume nor stroke volume index correlated with the corresponding thermodilution-based data. Even though non-invasive estimated continuous cardiac output monitoring consistently underestimated the corresponding thermodilution parameters, no trend analysis was achievable. Summarizing, we cannot suggest the use of non-invasive estimated continuous cardiac output monitoring as an alternative to transpulmonary thermodilution for cardiac output monitoring in pregnant patients undergoing minimally-invasive foetoscopic surgery for spina bifida.

## Introduction

Surgery for foetal spina bifida aperta in pregnant women represents a complex minimally-invasive procedure requiring interdisciplinary surgical and anaesthesiologic management. The prevention of maternal pulmonary oedema, adequate analgosedation of the mother and the unborn child, and foetal immobilization are of paramount importance for the procedure, which is performed under maternofoetal anaesthesia^1^. As the foetus is highly dependent on sufficient uteroplacental perfusion, which correlates with the mother´s blood pressure and cardiac output (CO), reliable haemodynamic assessment plays an integral role in the anaesthesiologic management of pregnant patients undergoing this procedure. Especially, CO monitoring as a surrogate for placental perfusion is at high interest to the anaesthesiologist^2,3^. Various approaches of haemodynamic monitoring under foetal surgery have been described ranging from absent CO monitoring to invasive CO measurement^1,4,5^. However, increasing evidence supports the importance of advanced haemodynamic monitoring including CO quantification in pregnant patients^6^. First, pregnancy leads to extensive changes to the maternal haemodynamic system which can hardly be precisely predicted under the anaesthetic conditions. Second, foetal surgery under general anaesthesia can induce rapid impairment of the maternal and foetal haemodynamic stability by changing intrathoracic (e.g., need for invasive ventilation) and intraabdominal pressure (e.g., partial amniotic carbon dioxide insufflation (PACI)) as well as decreasing the systemic vascular resistance (SVR)^1,5–7^. Since no guidelines for the haemodynamic management of foetal surgery exist, the recommendations of the International Working Group of Maternal Haemodynamics have to be considered^6^. The authors of these recommendations highlight the need for individual assessment of advanced haemodynamic monitoring (including CO measurement) in pregnant patients as well as individual definitions of thresholds of the measured haemodynamic parameters. In order to prevent critical intraoperative uteroplacental hypoperfusion and to detect perioperative pulmonary oedema, in our department pregnant patients undergoing surgery for foetal spina bifida aperta are provided with an advanced haemodynamic monitoring.

Several monitoring devices have been introduced for perioperative CO monitoring^8–10^. For pregnant patients undergoing major surgery, calibrated transpulmonary thermodilution (TPTD)-based haemodynamic monitoring offers two main advantages. First, it represents a widely accepted method for CO measurement. Second, it allows the quantification of extravascular lung water index (EVLWI) to ensure a rapid diagnosis of pulmonary oedema. Since pulmonary permeability increases during pregnancy, pregnant surgical patients are at a higher risk for the development of pulmonary oedema^1,11^. On the other hand, TPTD remains an invasive procedure requiring central venous and arterial access with a resulting risk for complications. Therefore, we opted to search for less-invasive techniques for CO monitoring in pregnant patients.

An existing estimated continuous cardiac output (esCCO) haemodynamic monitoring system (Nihon Kohden^®^, Tokyo, Japan) offers completely non-invasive quantification of CO using electrocardiogram (ECG) and plethysmography. The esCCO calculates the pulse wave transit time of the time from the end-diastolic phase (R-wave in ECG) to the capillary arrival time (plethysmography). Since the system uses morphometric parameters for calculation, no invasive calibration is necessary^12^. Its accuracy has been validated, but several studies were not able to offer a good correlation of the esCCO-based haemodynamic parameters and classical CO monitoring (e.g., echocardiography or thermodilution) in critically ill and perioperative patients^13–17^. Nevertheless, esCCO might be able to show a relative correlation of CO in a homogenous healthy patient cohort such as pregnant women.

Therefore, the purpose of this study was to evaluate the use of esCCO-based quantification of CO in comparison with the application of the well-established TPTD monitoring system in pregnant patients undergoing minimally-invasive foetoscopic surgery for spina bifida aperta.

## Results

### Patient´s characteristics

Between June 2016 and June 2018, 35 patients were enrolled, resulting in 199 measurement time points. All screened patients were included and none had a positive history for cardiovascular, respiratory, neurological, or metabolic disease. Baseline characteristics are shown in Table 1.

**Table 1:** Overview of patient characteristics and perioperative data.

Parameter	Median (Interquartile range)
**Patients characteristics**	
Age (years)	31.3 [27.5–35]
Gestation week	25 [25,26]
Body mass index (kg × m^−2^)	28.8 [24–29.4]
Hospital length of stay (days)	9.3 [4.5–9]
ICU length of stay (days)	1.3 [1]
Preoperative hemoglobin (g × l^−1^)	10 [8.9–10.7]
Preoperative hematocrit (g × l^−1^)	30.8 [27.8–33]
**Perioperative data**	
Systolic arterial blood pressure (mmHg)	113 [107–124]
Diastolic arterial blood pressure (mmHg)	59 [53–64]
Mean arterial blood pressure (mmHg)	79 [73–86]
Central venous pressure (mmHg)	12 [9–14]
Heart rate (beats × min^−1^)	77 [71–86]
SpO_2_ (%)	100 [99–100]
Temperature (°C)	36 [36.3–37]
EVLWI (ml × kg^−1^)	8.1 [7.2–9.2]
SVR (dyn × sec × cm^−5^)	617 [525–706]
SVRI (dyn × sec × cm^−5^ × m^−2^)	1166.5 [1024.3–1326]
**Mechanical ventilation data**	
Respiratory Rate (min^−1^)	15 [13.9–17]
Minute ventilation (l × min^−1^)	7.3 [6.4–8.1]
F_i_O_2_	0.6 [0.6]
PIP (cmH_2_O)	20 [18–24]
PEEP (cmH_2_O)	5 [5]
**Arterial blood gases**	
pH	7.37 [7.33–7.39]
P_a_O_2_ (mmHg)	280 [226–315]
P_a_CO_2_ (mmHg)	36.6 [33.7–38.4]
Lactate (mMol × l^−1^)	1.4 [1,2]
**Perioperative volume and catecholamine therapy**	
Intraoperative crystalloids (ml)	1410 [1075–1800]
Epinephrine dosage (µg × kg^−1^ × min^−1^)	0.02 [0.01–0.02]

Abbreviations: EVLWI = Extravascular lung water index; ICU = Intensive care unit; PEEP = Positive end-expiratory pressure; PIP = Positive inspiratory pressure; SVR = Systemic vascular resistance; SVRI = Systemic vascular resistance index.

### Comparison of esCCO-derived CO to pulse contour analysis

**Figure 1:**
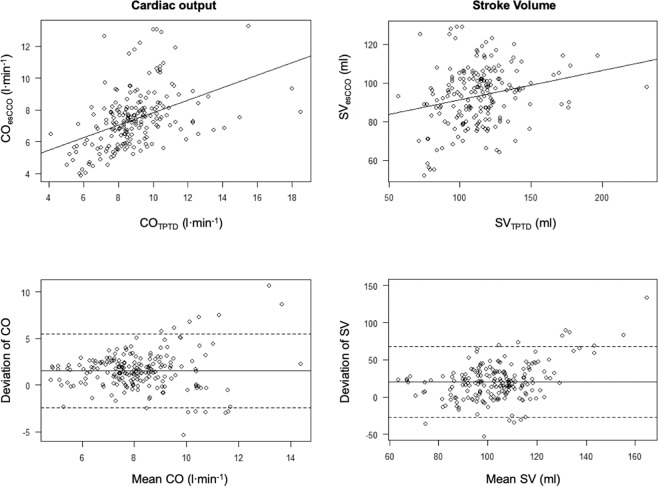
Scatterplots showing the correlation between CO_esCCO_ and CO_TPTD_ (upper left) and SV_esCCO_ and SV_TPTD_, respectively (upper right). Bland–Altman diagrams reveal the bias of both methods. The solid line shows the mean of the measured differences, while the dotted lines demonstrate the 95% limits of agreement (average difference ± 1.96 standard deviation of the difference).

**Figure 2:**
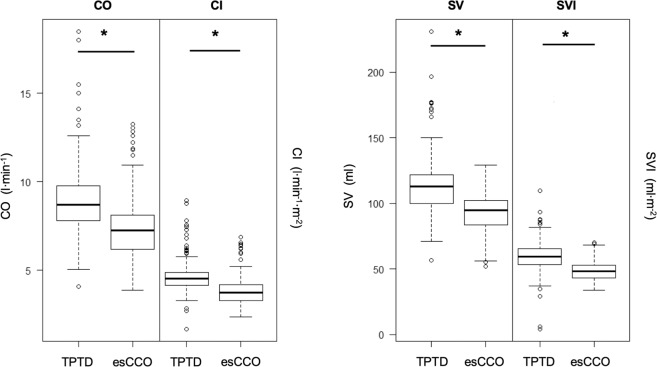
Box plots showing the distribution of all measured values of CO, CI, SV, and SVI. The black bar represents the median, while the box symbolizes the upper and lower quartile ranges. The whiskers show the 95% percentiles and outliners are represented by individual points. Asterisks indicate significant differences (*p* < 0.001).

**Figure 3:**
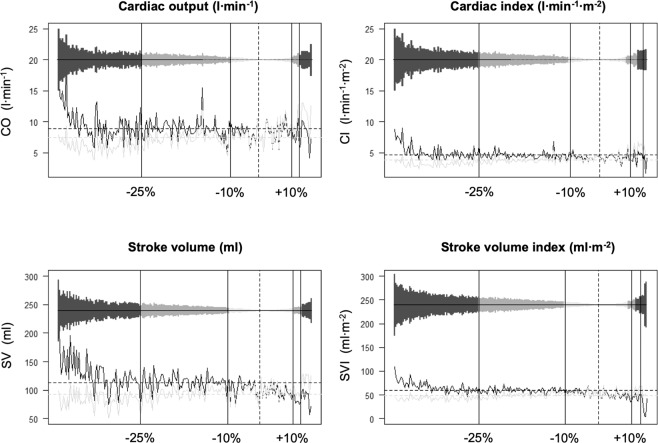
Diagram showing the range deviation of esCCO as compared with TPTD measurements. The bars represent the amount of difference (ordered from negative to positive) and the graphs depict the according measurements with esCCO (grey) and TPTD (black). The mean is shown as a dotted horizontal line and the vertical lines limit the range of the 10% or 25% deviation. Here, 0% to 10% deviation is presented as pale grey bars, 10% to 25% deviation is presented as grey bars, and more than 25% deviation is presented as dark grey bars, respectively.

CO_esCCO_ values showed a poor correlation with the CO_TPTD_ measurements (correlation coefficient: 0.44, R^2^: 0.19; CO_esCCO_: 7.4 [6.2–8.1] and CO_TPTD_: 8.9 [7.8–9.8]; *p* ≤ 0.001; Fig. 1), while cardiac index (CI) showed no correlation (correlation coefficient: 0.27, R^2^: 0.072; CI_esCCO_: 3.8 [3.3–4.2] and CI_TPTD_: 4.7 [4.2–4.9]; *p* ≤ 0.001). In accordance with this weak correlation, the concordance rates of CO and CI were also low (CO: 62.8% and CI: 64.8%, Supplement 1). Partial amniotic carbon dioxide insufflation (PACI) did neither influence the correlation of CO (CO before PACI: correlation coefficient: 0.58, R^2^: 0.34; CO_esCCO_: 6.8 [5.8–7.8], CO_TPTD_: 8.4 [7.4–9.4.]; *p* ≤ 0.001; CO during PACI: correlation coefficient: 0.39, R^2^: 0.16; CO_esCCO_: 7.3 [6.3–8.1], CO_TPTD_: 8.6 [7.7–9.7]; *p* ≤ 0.001) nor of CI in a relevantly manner (CI before PACI: correlation coefficient: 0.43, R^2^: 0.18; CI_esCCO_: 3.6 [3.1–4], CI_TPTD_: 4.4 [3.9–4.7]; *p* ≤ 0.01; CI during PACI: correlation coefficient: 0.25, R^2^: 0.06; CI_esCCO_: 3.7 [3.3–4.2], CI_TPTD_: 4.5 [4.2–4.9]; *p* ≤ 0.01). Of note, the esCCO analysis underestimated CO and CI consistently (Fig. 2). Only 24.2% of all CO measurements were within the tolerance of the 10% deviation (−10% to 0% deviation of CO = 11.1% of cases and 0–10% deviation of CO = 13.1% of cases; Fig. 3), while 37.7% of all CO measurements differed more than 25% between both methods (>25% downward deviation of CO = 33.2% of cases and >25% upward deviation of CO = 4.5% of cases; Fig. 3). Similar results were achievable regarding CI (>25% downward deviation of CI = 33.7% of cases, −10% to 0% deviation of CI = 11.1% of cases, 0–10% deviation of CI = 12.1% of cases, >25% upward deviation of CI = 4% of cases; Fig. 3).

### Comparison of esCCO-derived stroke volume to pulse contour analysis

Our results indicated that neither esCCO-derived stroke volume (SV) (correlation coefficient: 0.23, R^2^: 0.055; SV_esCCO_: 113.5 [100–122] and SV_TPTD_: 4.7 [4.2–4.9]; *p* ≤ 0.001) nor stroke volume index (SVI) (correlation coefficient: 0.044, R^2^: 0.0019; SVI_esCCO_: 48.7 [43.3–53] and SVI_TPTD_: 59 [53.5–65.3]; *p* ≤ 0.001; Fig. 1) correlated with the TPTD data. Furthermore, PACI did neither affect the correlations of SV nor of SVI (SV before PACI: correlation coefficient: 0.47, R^2^: 0.23; SV_esCCO_: 94 [83.5–102], SV_TPTD_: 112 [100.8–120.5]; *p* ≤ 0.001; SV during PACI: correlation coefficient: 0.2, R^2^: 0.04; SV_esCCO_: 95 [82–102], SV_TPTD_: 115 [98.5–125]; *p* ≤ 0.01; SVI before PACI: correlation coefficient: 0.17, R^2^: 0.03; SVI_esCCO_: 49 [44.5–55], SVI_TPTD_: 57.7 [51.3–63.9]; *p* = 0.34; SVI during PACI: correlation coefficient: −0.02, R^2^: <0.001; SVI_esCCO_: 48 [43–53], SVI_TPTD_: 60 [53.9–66.1]; *p* = 0.83). Concordance rates were also poor (SV: 53%, SVI: 50.7%, Supplement 1). Analogous to the CO measurements, SV- and SVI_esCCO_ were underestimated significantly (Fig. 2). Additionally, 25.7% of both (SV and SVI) deviated within the 10% limit (−10% to 0% deviation of SV = 12.6% of cases, 0–10% deviation of SV = 13.1% of cases, −10% to 0% deviation of SVI = 12.6% of cases, and 0–10% deviation of SVI = 13.1% of cases; Fig. 3), while 36.7% of all SV_esCCO_ and 38.7% of SVI_esCCO_ measurements diverged more than 25% as compared with SV- and SVI_TPTD_ data (>25% downward deviation of SV = 32.7% of cases, >25% upward deviation of SV = 4% of cases, >25% downward deviation of SVI = 33.7% of cases, and >25% upward deviation of SV = 5% of cases; Fig. 3).

## Discussion

This study evaluated the validity of the non-invasive esCCO monitoring for the assessment of CO and SV as well as their indices in pregnant patients undergoing minimally-invasive foetoscopic surgery for spina bifida. As such, a healthy and homogenous cohort of pregnant patients who underwent minimally-invasive foetoscopic surgery for spina bifida of their unborn child was investigated. Our study found that esCCO-derived parameters did not correlate with the results of the pulse contour analysis. Only CO_esCCO_ showed a weak correlation with CO_TPTD_. Analogous results were found in a subanalysis investigating the influence of PACI on the performance of esCCO. Overall, esCCO underestimated CO, CI, SV, and SVI consistently. Supposing a tolerance of 10% deviation to the established TPTD, esCCO failed to achieve reliable results.

The assessment of CO in perioperative medicine remains part of advanced haemodynamic anaesthesiologic management^10,18^. The gold standard of CO quantification remains the catheterization of the pulmonary artery with consecutive CO measurement based on Fick´s method. However, this method is considered more invasive in comparison with TPTD analysis and is therefore only recommended in critically ill pregnant patients^6,11^. Several studies have shown that TPTD-based CO quantification remains also under difficult haemodynamic conditions comparable to pulmonal artery catheter (PAC) measurement^6,11,19–22^. While early validation studies showed a good concordance of PAC and TPTD-deriving CO measurements, later studies revealed a slight overestimation of TPTD-based CO quantification^19–21,23^. Two further studies supported the sufficient concordance of TPTD and PAC-based CO quantification but identified also limited accuracies in the quantification of varying CO trends^23,24^. For this reason, the recommendations of the International Working Group of Maternal Haemodynamics highlight that still no optimal device for CO quantification has been introduced^6^. However, TPTD offers reliable perioperative CO and EVLWI quantification with a low risk of complications and is therefore incorporated in the management of minimally-invasive foetoscopic surgery for spina bifida aperta in our department^1^. Nevertheless, it has to be emphasized that TPTD-based pulse contour analysis is not well investigated in pregnant patients. Up today, three different pulse contour analysis devices have been evaluated in pregnancy: the PiCCO^®^, LiDCO^®^ (LiDCO, London, UK), and VolumeView^®^ (Edwards Lifesciences, Irvine, USA) systems. While LiDCO^®^ has been validated in pregnant patients and is less invasive than the PiCCO^®^ and the VolumeView^®^ system, it remains contraindicated in the first trimester due to potential adverse effects on the foetal neurological development making it hardly suitable for foetal surgery occurring in this period of pregnancy. Furthermore, other than the PiCCO^®^ and VolumeView^®^ system, LiDCO^®^ does not offer the quantification of EVLWI which is particularly important in foetal surgery for the diagnosis of perioperative pulmonary oedema^1^. Regarding pregnant patients, both, the PiCCO^®^ and VolumeView^®^ system, have only been evaluated in a small amount of studies^6,25–27^. For this reason, the International Working Group of Maternal Haemodynamics recommends (independently of foetal surgery) further studies investigating the safety and benefit of pulse contour analysis in pregnant patients^6^.

While most devices are invasive (e.g., pulse contour analysis, transoesophageal echocardiography) or not practicable in the operating room (e.g., transthoracic echocardiography), esCCO offers the possibility of non-invasive CO quantification. Several studies have attested a sufficient validity of CO_esCCO_ and SV_esCCO_ and their indices in heterogenous patients cohorts^28–30^. However, other research suggested controversial results indicating a weak correlation and a general underestimation of esCCO parameters with established methods such as TPTD or echocardiography^12–14,31,32^. One possible explanation for the low correlation of esCCO to TPTD analysis might be the underlying technology. esCCO uses the pulse wave transit time in order to calculate CO, which is negatively correlated with the cardiac SV and which depends highly on patient variables such as age, sex, weight, and height^33^. These variables do not reflect the pregnancy-induced changes of the circulatory system, which might explain the low validity of the esCCO results. Several haemodynamic changes occur during pregnancy. First, blood volume and heart rate increase, leading to an elevated CO by up to 50% as well as physiological anaemia, which reaches a maximum in the late second trimester. Second, the growing uterus provokes increased intra-abdominal pressure on the inferior vena cava, causing a decrease in venous return from the lower body and abdomen. Especially in the supine position, these effects may promote a reduced cardiac preload, leaving patients at risk for acute intraoperative hypotension with consecutive uteroplacental hypoperfusion^2^. Furthermore, pulse-wave transit time is based on perfusion time through the peripheral arteries and therefore highly depends upon peripheral arterial resistance. Since the study cohort here consisted of pregnant patients in the second trimester, peripheral arterial resistance was decreased as compared with in nonpregnant patients, indicated by a significant reduced SVR. Studies investigating esCCO in critically ill patients described similar results and proposed that changes in SVR might explain the poor correlation of esCCO and echocardiography^12,31^. Analogues to the findings of this study, Biais *et al*. reported an underestimation of actual CO through esCCO associated with a reduced SVR^12^. Due to the physiologic arterial vasodilatation that occurs in pregnancy, SVR was even significantly lower in our cohort than in critically ill patients, indicating a further possible explanation for the weak performance of esCCO^34^. However, these data do not support Yamada *et al*.´s conclusion that SVR only slightly affects the accuracy of this method^35^. From our point of view, pregnancy-induced low SVR might have caused the weak correlation of esCCO and TPTD parameters in our study cohort. Since the uteroplacental perfusion is dependent of the blood pressure and CO, an underestimation of CO displays a potentially critical problem regarding the anaesthetic monitoring in pregnant patients undergoing foetal surgery. TPTD-based measurements may also be vulnerable to changes in SVR, for this reason we chose to calibrate before each esCCO quantification. Therefore, we assume that the TPTD data are reliable.

Despite TPTD monitoring being well-established as a tool in minimally-invasive foetoscopic surgery of foetal spina bifida in our department, we searched for a non-invasive method for CO measurement. In this context, CO represents together with other vital parameters a surrogate for the perfusion of the uteroplacental unit and is therefore of major concern in the context of anaesthesiologic management. Since esCCO failed to correlate with measurements of the pulmonary thermodilution analysis and does not offer the possibility of quantifying SVR or EVLWI, we cannot recommend the use of esCCO for CO monitoring in pregnant patients undergoing this minimally-invasive procedure.

Our study offers one main limitation of note, since TPTD was used as the reference method for the evaluation of esCCO. Despite our department being highly experienced with the use of TPTD in pregnant patients, this approach’s use is still not well-evaluated for application in this special patient cohort. The decreased peripheral resistance might also affect the TPTD measurements. However, the similar LiDCO^®^ system is also based on pulse contour analysis and is validated for pregnant patients^6,36^. Due to the elevated risks, methodical comparisons of pulmonary artery catheterization and TPTD in pregnant patients do not exist, but data of non-pregnant patients and the significant experience of TPTD in pregnant patients allow its use in patients undergoing minimally-invasive foetoscopic surgery for spina bifida^6,36,37^. Furthermore, even though the anaesthetic management of fetoscopic surgery is highly standardized and measurements were only performed in stable haemodynamic situations, it cannot be ruled out that other factors such as the ventilation strategy or changes of the intravascular fluid status might have influenced the performance of the esCCO device. Last, two studies performed cross-comparisons of various methods for CO quantification (PAC, TPTD, and bioreactance measurements) in cardiac surgical patients and showed limited trending accuracies between the devices. For this reason, future validation studies should use more than two methods for CO quantification^23,24^.

Summarizing, we used for the comparison of esCCO-derived CO and SV parameters the well-established calibrated pulse contour analysis–based monitoring system. While CO_esCCO_ correlated only weakly with CO_TPTD_, CI_esCCO_, SV_esCCO_, and SVI_esCCO_ did not correlate at all with the corresponding TPTD parameters. Furthermore, esCCO consistently underestimated the TPTD parameters, and no trend analysis was achievable due to a high range of deviations. Therefore, we cannot support the use of esCCO for CO monitoring in pregnant patients undergoing minimally-invasive foetoscopic surgery for foetal spina bifida.

## Methods

### Study design and patient enrolment

This noninterventional, explorative study was approved by the local ethics committee (Justus-Liebig-University, Giessen, Germany, trial code 123/15) and was performed prospectively according to the approved study design. However, it was registered retrospectively in the German clinical trials register (registration number: DRKS00008769). The study methods and results are presented in accordance with the Strengthening the Reporting of Observational Studies in Epidemiology (STROBE) guidelines and the declaration of Helsinki.

All eligible patients were enrolled at the university hospital of Giessen and signed an informed consent form prior to study inclusion. Pregnant patients of legal age undergoing minimally-invasive foetoscopic surgery for spina bifida were included, while patients with cardiac diseases such as atrial fibrillation or cardiomyopathy and/or the presence of a cardiac pacemaker were excluded.

### Anaesthesiologic management

The anaesthesiologic management was performed as already published^1^. Briefly, patients fasted six hours prior to surgery and took aspiration prophylaxis (150 mg of ranitidine orally). Before the induction of anaesthesia, patients received intravenous antibiotic prophylaxis (600 mg of clindamycin and 120 mg of gentamicin) and tocolysis (intravenous bolus of 6.75 mg of atosiban followed by an infusion of 9 mg/h for 24 hours). Rapid-sequence induction of anaesthesia was performed with fentanyl (2–5 µg × kg^−1^), thiopental (5 mg × kg^−1^), and rocuronium (1 mg × kg^−1^). Monitoring included ECG, invasive blood pressure, plethysmography, pulse contour analysis, bladder temperature, capnography, blood gas analysis, gas monitoring, bispectral index, relaxometry, and umbilical as well as foetal Doppler sonography. Anaesthesia was maintained with desflurane [Minimal alveolar concentration: 0.5–0.7 (~3.0–4.2 vol%)], repeated intravenous boli of cis-atracurium (0.02–0.05 mg × kg^−1^), and continuous remifentanil infusion (0.05–0.1 μg × kg^−1^ × min^−1^). Medications were adjusted to meet maternal and foetal hypnotic and analgesic requirements. Mechanical ventilation was adjusted to a tidal volume of 6 to 8 ml × kg^−1^ (ideal estimated body weight) and a positive end-expiratory pressure of 5 cmH_2_O, and ventilation frequency was established according to end-tidal and arterial carbon dioxide. Volume management was performed according to the TPTD data. If necessary, patients received crystalloid boli and adrenalin infusions (starting dosage: 0.1 μg × kg^−1^ × min^−1^). Anaesthesiologic management targets included the avoidance of hypercapnia, sufficient oxygenation, stable haemodynamic parameters, adequate placental perfusion, intrathoracic blood volume of less than 850 ml × m^−2^ and extravascular lung water index of 10 ml × kg^−1^ or less. Patients were extubated in the operating theatre, transmitted to the surgical intermediate care ward, and surveilled until the first surgical day.

### Pulse contour analysis and thermodilution measurements

Arterial access was established in the femoral artery with a 4-French thermistor-tipped artery catheter (Pulsiocath PV2014L22-A; Pulsion Medical Systems, Munich, Germany), while a central venous catheter was implemented in the right internal jugular vein (Arrow^®^; Teleflex Medical, Kernen, Germany). Central venous pressure and pulse contour analysis (Pulse Contour CO^®^ and PiCCO^®^; Pulsion Medical Systems, Feldkirchen, Germany) were measured continuously. For the pulse contour analysis, proper calibration via TPTD was performed with boli of cold saline according to the manufacturer´s instructions^38,39^. Morphometric data were entered before calibration. Calibration was performed after implementation of the PiCCO^®^ system, directly before esCCO measurements, and after admission to the intermediate care ward. Recorded values included SV (SV_TPTD_, ml), SVI (SVI_TPTD_, ml × m^−2^), CO (CO_TPTD_, l × min^−1^), CI (CI_TPTD_, l × min^−1^ × m^−2^), EVLWI (ml × kg^−1^), SVR (dyn × sec × cm^−5^), and systemic vascular resistance index (SVRI, dyn × sec × cm^−5^ × m^−2^). CO was measured priorly to the begin of the foetoscopic surgery under stable conditions. The obtained CO was used as a referral value for the following measurements. With regard to other haemodynamic parameters such as maternal blood pressure, urine production, and foetal doppler signals, CO trending was considered for haemodynamic management. No general relative threshold of CO trending was defined.

### esCCO

esCCO was measured with the PVM-2703 K bedside monitor (Nihon Kohden, Tokyo, Japan) following the correct input of individual patient parameters. The blood pressure cuff and plethysmography sensor were installed according to the manufacturer´s instructions. CO_esCCO_ (l × min^−1^) was calculated continuously by the monitor algorithm as already published^12,33^. SV_esCCO_ (ml), SVI_esCCO_ (ml × m^−2^) and CI_esCCO_ (l × min^−1^ × m^−2^) were documented. Time points of haemodynamic measurements (TPTD and esCCO) depended upon stable haemodynamic status (i.e., stable sinus rhythm, blood pressure, and plethysmography curve for at least two minutes) and were chosen after the anaesthesiologist´s assessment.

### Data processing and statistical analysis

Clinical data of the anaesthesiologic management as well as the data of the intermediate care ward were obtained from the hospital´s patient data management systems Narco- and ICU-Data^®^ (IMESO^®^ GmbH, Giessen, Germany). In comparison with the corresponding TPTD data, a difference of 10% of the esCCO-derived CO, CI, SV, and SVI measurements was defined as a tolerable deviation. Descriptive analysis was performed for demographic data and clinical characteristics. All variables are presented as medians with interquartile ranges, while categorical variables are presented as numbers and percentages. Statistical significance was tested by applying a two-sample t-test and defined as a p-value of 0.05 or less. The association between measurements was evaluated by correlation test, with calculation of the Pearson correlation coefficient. Measurements were assessed by Bland–Altman analysis^40^. Statistical analysis was performed using *R* Statistics, version 3.5.2 (www.r-project.org; The R Foundation, Vienna, Austria).

## Supplementary information

Supplement 1.

## References

[1] Arens C (2017). Anesthetic Management for Percutaneous Minimally Invasive Fetoscopic Surgery of Spina Bifida Aperta: A Retrospective, Descriptive Report of Clinical Experience. Anesth. Analg..

[2] Reitman E, Flood P (2011). Anaesthetic considerations for non-obstetric surgery during pregnancy. Br. J. Anaesth..

[3] Valdés G, Corthorn J (2011). Review: The angiogenic and vasodilatory utero-placental network. Placenta.

[4] Ferschl M, Ball R, Lee H, Rollins MD (2013). Anesthesia for in utero repair of myelomeningocele. Anesthesiology.

[5] Hoagland MA, Chatterjee D (2017). Anesthesia for fetal surgery. Paediatr. Anaesth..

[6] Bijl RC (2019). Methods and considerations concerning cardiac output measurement in pregnant women: recommendations of the International Working Group on Maternal Hemodynamics. Ultrasound Obstet. Gynecol..

[7] Ziemann M (2018). Partial amniotic carbon dioxide insufflation (PACI) during minimally invasive fetoscopic interventions on fetuses with spina bifida aperta. Surg. Endosc..

[8] Saugel B, Vincent JL, Wagner JY (2017). Personalized hemodynamic management. Curr. Opin. Crit. Care.

[9] Saugel B, Cecconi M, Wagner JY, Reuter DA (2015). Noninvasive continuous cardiac output monitoring in perioperative and intensive care medicine. Br. J. Anaesth..

[10] Vincent JL (2015). Perioperative cardiovascular monitoring of high-risk patients: A consensus of 12. Crit. Care.

[11] Monnet X, Teboul JL (2017). Transpulmonary thermodilution: Advantages and limits. Crit. Care.

[12] Biais M, Berthezène R, Petit L, Cottenceau V, Sztark F (2015). Ability of esCCO to track changes in cardiac output. Br. J. Anaesth..

[13] Fischer MO (2014). The diagnostic accuracy of estimated continuous cardiac output compared with transthoracic echocardiography. Can. J. Anesth..

[14] Feissel M (2015). Pulse wave transit time measurements of cardiac output in septic shock patients: A comparison of the estimated continuous cardiac output system with transthoracic echocardiography. PLoS One.

[15] Smetkin AA (2017). Estimated continuous cardiac output based on pulse wave transit time in off-pump coronary artery bypass grafting: a comparison with transpulmonary thermodilution. J. Clin. Monit. Comput..

[16] Suzuki T (2018). Cardiac output and stroke volume variation measured by the pulse wave transit time method: a comparison with an arterial pressure-based cardiac output system. J. Clin. Monit. Comput..

[17] Terada T, Maemura Y, Yoshida A, Muto R, Ochiai R (2014). Evaluation of the estimated continuous cardiac output monitoring system in adults and children undergoing kidney transplant surgery: A pilot study. J. Clin. Monit. Comput..

[18] Chong MA, Wang Y, Berbenetz NM, McConachie I (2018). Does goal-directed haemodynamic and fluid therapy improve peri-operative outcomes?: A systematic review and meta-analysis. Eur. J. Anaesthesiol..

[19] Della Rocca G, Costa MG, Pompei L, Coccia C, Pietropaoli P (2002). Continuous and intermittent cardiac output measurement: Pulmonary artery catheter versus aortic transpulmonary technique. Br. J. Anaesth..

[20] Sakka SG, Reinhart K, Meier-Hellmann A (1999). Comparison of pulmonary artery and arterial thermodilution cardiac output in critically ill patients. Intensive Care Med..

[21] Friesecke S, Heinrich A, Abel P, Felix SB (2009). Comparison of pulmonary artery and aortic transpulmonary thermodilution for monitoring of cardiac output in patients with severe heart failure: Validation of a novel method. Crit. Care Med..

[22] Sakka SG, Reuter DA, Perel A (2012). The transpulmonary thermodilution technique. J. Clin. Monit. Comput..

[23] Lamia B, Kim HK, Severyn DA, Pinsky MR (2018). Cross-comparisons of trending accuracies of continuous cardiac-output measurements: pulse contour analysis, bioreactance, and pulmonary-artery catheter. J. Clin. Monit. Comput..

[24] Hadian M, Kim HK, Severyn DA, Pinsky MR (2010). Cross-comparison of cardiac output trending accuracy of LiDCO, PiCCO, FloTrac and pulmonary artery catheters. Crit. Care.

[25] Matsota P (2015). The effect of 0.5 L 6% hydroxyethyl starch 130/0.42 versus 1 L Ringer’s lactate preload on the hemodynamic status of parturients undergoing spinal anesthesia for elective cesarean delivery using arterial pulse contour analysis. J. Anesth..

[26] Auler JOC (2010). Clinical evaluation of the flotrac/Vigileo system for continuous cardiac output monitoring in patients undergoing regional anesthesia for elective cesarean section: a pilot study. Clinics (Sao Paulo)..

[27] Brogly Nicolas, Schiraldi Renato, Puertas Laura, Maggi Genaro, Yanci Eduardo Alonso, Maldonado Ever Hugo Martinez, Arévalo Emilia Guasch, Rodríguez Fernando Gilsanz (2016). Pulse contour analysis calibrated by Trans-pulmonar thermodilution (Picco Plus®) for the perioperative management of a caesarean section in a patient with severe cardiomyopathy. Brazilian Journal of Anesthesiology (English Edition).

[28] Ishihara H (2012). The ability of a new continuous cardiac output monitor to measure trends in cardiac output following implementation of a patient information calibration and an automated exclusion algorithm. J. Clin. Monit. Comput..

[29] Yamada T (2012). Multicenter study verifying a method of noninvasive continuous cardiac output measurement using pulse wave transit time: A comparison with intermittent bolus thermodilution cardiac output. Anesth. Analg..

[30] Tsutsui M (2013). Pulse wave transit time measurements of cardiac output in patients undergoing partial hepatectomy: A comparison of the esCCO system with thermodilution. Anesth. Analg..

[31] Bataille B (2012). Comparison of esCCO and transthoracic echocardiography for non-invasive measurement of cardiac output intensive care. Br. J. Anaesth..

[32] Thonnerieux M (2015). The ability of esCCO^TM^ and ECOM^TM^ Monitors to measure trends in cardiac output during alveolar recruitment maneuver after cardiac surgery: A comparison with the pulmonary thermodilution method. Anesth. Analg..

[33] Sugo Y (2010). A novel continuous cardiac output monitor based on pulse wave transit time. Conf. Proc…. Annu. Int. Conf. IEEE Eng. Med. Biol. Soc. IEEE Eng. Med. Biol. Soc. Annu. Conf..

[34] Heesen Michael, Klimek Markus (2016). Nonobstetric anesthesia during pregnancy. Current Opinion in Anaesthesiology.

[35] Yamada T (2012). Multicenter Study Verifying a Method of Noninvasive Continuous Cardiac Output Measurement Using Pulse Wave Transit Time. Anesth. Analg..

[36] Dyer RA (2011). Comparison between pulse waveform analysis and thermodilution cardiac output determination in patients with severe pre-eclampsia. Br. J. Anaesth..

[37] Cornette J (2017). Validation of maternal cardiac output assessed by transthoracic echocardiography against pulmonary artery catheterization in severely ill pregnant women: prospective comparative study and systematic review. Ultrasound Obstet. Gynecol..

[38] Neumann P (1999). Extravascular lung water and intrathoracic blood volume: double versus single indicator dilution technique. Intensive Care Med..

[39] Sakka SG (2000). Assessment of cardiac preload and extravascular lung water by single transpulmonary thermodilution. Intensive Care Med..

[40] Critchley LAH (2017). Meta-analyses of Bland-Altman-style cardiac output validation studies: good, but do they provide answers to all our questions?. Br. J. Anaesth..

