# Supplementary material for: Evaluation of pulse wave transit time analysis for non-invasive cardiac output quantification in pregnant patients
Source: Sci Rep. 2020 Feb 5;10:1857. doi: 10.1038/s41598-020-58910-x (PMC7002624; doi:10.1038/s41598-020-58910-x)
Supplement: Supplementary file 1 — Supplement 1. [file 41598_2020_58910_MOESM1_ESM.docx]

**Evaluation of pulse wave transit time analysis for non-invasive cardiac output quantification in pregnant patients**

Emmanuel Schneck^1*^, Pascal Drubel^1^, Rainer Schürg^1^, Melanie Markmann^1^, Thomas Kohl^2^, Michael Henrich^3^, Michael Sander^1^, Christian Koch^1^

^1^Justus Liebig University of Giessen, Department of Anesthesiology, Operative Intensive Care Medicine and Pain Therapy, Rudolf-Buchheim-Strasse 7, 35392 Giessen, Germany.

*^2^German Center for Fetal Surgery & Minimally* Invasive Therapy (DZFT), University Hospital of Mannheim, *Theodor-Kutzer-Ufer 1-3, 68167 Mannheim, Germany*

*^3^Department of Anesthesiology and Intensive Care Medicine, St. Vincentius Clinics, Suedendstrasse 32, 76137 Karlsruhe, Germany*

**Supplement 1**

## Supplement 1

| **CO** | | **Δ esCCO** | | **%** | | **Δ esCCO** | |  | **CI** | | **Δ esCCO** | | **%** | | **Δ esCCO** | |
| --- | --- | --- | --- | --- | --- | --- | --- | --- | --- | --- | --- | --- | --- | --- | --- | --- |
|  |  | >0 | <0 |  |  | >0 | <0 |  |  |  | >0 | <0 |  |  | >0 | <0 |
| **Δ TPTD** | >0 | 23 | 5 | **Δ TPTD** | >0 | 48,9% | 10,6% |  | **Δ TPTD** | >0 | 24 | 4 | **Δ TPTD** | >0 | 51,1% | 8,5% |
|  | <0 | 7 | 12 |  | <0 | 14,9% | 25,5% |  |  | <0 | 8 | 11 |  | <0 | 17,0% | 23,4% |
|  |  |  |  |  |  |  |  |  |  |  |  |  |  |  |  |  |
| **SV** | | **Δ esCCO** | | **%** | | **Δ esCCO** | |  | **SV** | | **Δ esCCO** | | **%** | | **esCCO** | |
|  |  | >0 | <0 |  |  | >0 | <0 |  |  |  | >0 | <0 |  |  | >0 | <0 |
| **Δ TPTD** | >0 | 21 | 19 | **Δ TPTD** | >0 | 28,4% | 25,7% |  | **Δ TPTD** | >0 | 16 | 4 | **Δ TPTD** | >0 | 47,1% | 11,8% |
|  | <0 | 12 | 22 |  | <0 | 16,2% | 29,7% |  |  | <0 | 4 | 10 |  | <0 | 11,8% | 29,4% |

**Supplement 1. Concordance of both monitors**. Concordance has been evaluated from Four Quadrant Plots and is given as absolute number of cases with same direction (both >0 / green or both <0 / red) as well as in percentage of total number of cases.
